# Integrated Gene–Metabolite Network Analysis Identifies Pathways Associated with Immune Regulation and Metabolic Remodeling in Subfertile Beef Heifers

**DOI:** 10.3390/genes17080889

**Published:** 2026-07-29

**Authors:** Priyanka Banerjee, Rachel Phillips, Anna G. Holliman, Soren P. Rodning, Wellison J. S. Diniz, Paul W. Dyce

**Affiliations:** Department of Animal Sciences, College of Agriculture, Auburn University, Auburn, AL 36849, USA; rah0057@auburn.edu (R.P.); agh0112@auburn.edu (A.G.H.); rodnisp@auburn.edu (S.P.R.); wzd0027@auburn.edu (W.J.S.D.)

**Keywords:** beef heifer, gene–metabolite integration, metabolome, network analysis, reproductive efficiency, subfertility, transcriptome

## Abstract

Background/Objectives: Reproductive inefficiency remains a major contributor to heifer culling and reduced herd longevity in beef systems. This study examined the molecular basis of fertility by analyzing granulosa cells and follicular fluid from Angus–Simmental crossbred heifers classified as fertile or subfertile. Methods: Granulosa cells and follicular fluid were collected for RNA sequencing and metabolomic analysis. Differential expression, network, and gene–metabolite integration analyses identified genes, metabolites, and pathways associated with fertility differences between groups. Results: We identified 90 differentially expressed genes from the granulosa cells, including *CXCL12* and *HOXD* family members, *CALCRL*, *DNER*, *GADD45G*, immune-related genes, and nine metabolites differentially abundant in follicular fluid, including parabanic acid, pimelic acid, α-tocopherol, glycine, and arachidonic acid. Network analysis revealed extensive rewiring in the network from the subfertile heifers for both genes and metabolites as compared to the fertile heifers. The gene–metabolite integration revealed a high degree of connectivity between arachidonic acid and α-tocopherol, correlating with immune and signaling genes, and between *CXCL12* and carbohydrate intermediates. Pathway over-representation analysis highlighted carbohydrate and purine metabolism in fertile heifers, and aminoacyl-tRNA biosynthesis, alanine/aspartate/glutamate metabolism, and Hippo signaling in the subfertile heifers. ABC transporters, ferroptosis, mineral absorption, and glyoxylate/dicarboxylate metabolism were common pathways identified in both groups. Integrative granulosa-follicular fluid analysis revealed coordinated gene–metabolite rewiring in subfertility, including functional candidates (*CXCL12*, *CALCRL*, *DNER*; α-tocopherol, arachidonic acid, fucose, parabanic acid) and fertility-associated pathways. Conclusions: These signatures provide novel targets and pathways underlying beef heifer fertility.

## 1. Introduction

Reproductive failure remains one of the primary reasons for culling breeding-age heifers, ultimately reducing herd longevity and producer profitability [1]. Although management practices, health status, and genetics strongly influence reproductive outcomes, ovarian function and oocyte quality are critical determinants of fertility in beef heifers [1,2,3]. The oocyte depends on granulosa cells and follicular fluid for maturation and developmental competence [4]. Granulosa cells provide metabolic support and maintain meiotic arrest, while follicular fluid supplies essential nutrients, hormones, growth factors, and antioxidants that collectively create a supportive microenvironment for oocyte health and fertilization potential [4].

Advances in genomic technologies have expanded our understanding of granulosa cell function and the molecular mechanisms within the follicular microenvironment that regulate fertility. Both granulosa cells and follicular fluid modulate the environment required for successful oocyte maturation and early embryonic development. RNA-Seq technology has enabled high-resolution profiling of granulosa cell transcriptomes, identifying differentially expressed genes associated with reproductive success. For instance, single-cell transcriptomics revealed an increased proportion of granulosa cells in pregnant heifers [5]. Microarray-based studies have also identified genes (*IGF2*, *RELN*, *KCNJ8*, *NRP1*, *ANKRD1*) and transcription factors (*STAT1*, *CEBPA*, *TP53*, *CREBBP*, and *SMAD4*) associated with high oocyte competence in bovines [6]. Similarly, granulosa cell transcriptome profiling following ovarian super-stimulation identified upregulation of *NTS*, *FOS*, *THBS1*, and *IGFBP1* along with downregulation of *NRP1*, *ANKRD43*, and *LTF* in beef cows [7]. Persistent transcriptomic alterations have also been observed in granulosa cells under conditions such as metritis [8], heat stress [9], differences in antral follicle size [10], superovulation, and aging [11].

Likewise, follicular fluid plays a critical role in regulating the microenvironment for oocyte growth and maturation [4]. It is important to note that there is a direct link between the follicular fluid and granulosa cells, as granulosa cells play a role in generating the osmotic gradient required for antrum formation through their production of hyaluronan, amongst other molecules [12]. The granulosa also contribute to the metabolic composition of follicular fluid, which can serve as a biomarker of oocyte quality. For example, increased retinol levels in follicular fluid promoted blastocyst formation but impaired attachment [13], while fatty acid supplementation affected the follicular environment and blastocyst production [14]. Metabolomic studies of follicular fluid have further identified biomarkers distinguishing high- and low-fertility cows [15,16,17]. Despite these advances, most studies have focused on single biological layers, often relying on differential expression analyses that overlook complex gene–metabolite interactions and their coordinated regulation across regulatory layers. Thus, a systems-level approach is needed to capture the interplay between transcriptomic and metabolic networks underlying fertility. To date, there is limited understanding of the integrated relationship between fertility potential, granulosa cell gene expression, and follicular fluid metabolite abundance. This study’s novelty lies in an integrative, network-based approach to identify coordinated gene–metabolite interactions associated with fertility. We hypothesized that heifer fertility is, at least in part, governed by the co-expression and coordinated interactions of granulosa-derived transcripts and follicular fluid metabolites. We integrated granulosa cell transcriptomes with follicular fluid metabolomes using a network-based approach to identify candidate genes, metabolites, and gene–metabolite pairs. Additionally, we identified fertility-associated biological pathways emerging from gene–metabolite interactions.

## 2. Materials and Methods

### 2.1. Animal Handling and Phenotype Collection

All animal procedures were approved by the Auburn University Institutional Animal Care and Use Committee (IACUC; protocol #2021-3968 and #2022-4096) and were conducted following the Planning Research and Experimental Procedures on Animals: Recommendations for Excellence (PREPARE) and Animal Research: Reporting of In Vivo Experiments (ARRIVE) guidelines.

Crossbred (Angus–Simmental) heifers were sourced from two Auburn University research stations. Initially, 48 heifers were housed at E.V. Smith Research Station, Shorter, AL, while 75 heifers were housed at the College of Veterinary Medicine North Auburn Beef Unit (CVM NABU), Auburn, AL. In both locations, heifers were developed on pasture forages with hay supplementation. Approximately 30 days before the late November breeding season, when heifers were ~400 days of age, pubertal status was assessed using reproductive tract scoring (RTS, scale 1–5; 1 = prepubertal, infantile; 5 = pubertal, luteal phase), as described [18]. During the breeding season, estrous synchronization was performed using a fixed-time artificial insemination (AI) protocol (7-Day CO-Synch + CIDR). Heifers received an intramuscular (IM) injection of 100 µg gonadotropin-releasing hormone (GnRH; Cystorelin, Merial Inc., Duluth, GA, USA) along with an intravaginal controlled internal drug-releasing device (CIDR) containing 1.38 g of progesterone (Eazi-Breed CIDR, Zoetis Inc., Kalamazoo, MI, USA). After seven days, CIDRs were removed, and heifers received an IM injection of 25 mg dinoprost tromethamine (Lutalyse, Zoetis Inc., Kalamazoo, MI, USA). Approximately 54 ± 2 h post-CIDR removal, heifers were artificially inseminated with cryopreserved semen from two commercially available Angus sires with proven fertility. Pregnancy status was determined 42 days post-AI via transrectal ultrasonography by a trained veterinarian.

Heifers confirmed pregnant by AI (*n* = 8) were administered 2 mL Lutalyse (Dinoprost tromethamine, IM) for pregnancy termination and classified as fertile. Heifers that did not conceive to AI were subsequently exposed to two proven fertile bulls that had successfully passed a breeding soundness exam 30 days prior to use, for a 60-day natural breeding season. Those that failed to conceive after AI and natural breeding (*n* = 5) were classified as subfertile. Following the final pregnancy diagnosis, thirteen heifers (8 fertile, 5 subfertile) were consolidated and housed at the Auburn University Beef Teaching Center (Auburn, AL, USA). Fertile heifers were those that conceived from AI, while subfertile heifers were defined as those that failed to conceive after AI and an additional 60-day period of natural breeding (non-pregnant group). There were no phenotypic differences (body condition score, RTS, or body weight) or physiological signs of infection between the two groups of heifers. All heifers were harvested at the Auburn University Lambert-Powell Meats Laboratory. Sample collection occurred across three harvest dates.

### 2.2. Sample Collection

Ovaries were collected from heifers at harvest and transported to the lab in coolers containing ice. Ovaries were washed at room temperature with 0.8% saline buffer. Follicular fluid was aspirated from follicles sized 2–6 mm from each heifer using 18-gauge short-bevel needles and sterile 10 mL syringes. The aspirate from each heifer was placed in separate conical-bottomed 15 mL polypropylene centrifuge tubes. Granulosa cells and cumulus-oocyte complexes (COCs) were allowed to settle for 8–10 min in the 15 mL tube. After removal of COCs, the follicular fluid with granulosa cells was centrifuged (500× *g* for 5 min at room temperature) to pellet granulosa cells. Follicular fluid was collected in separate microtubes and stored at −80 °C for a metabolomics study. The granulosa cell pellet was resuspended in DMEM-F12 (Gibco, Thermo Fisher Scientific, Billings, MT, USA) with 10% fetal bovine serum (FBS) and 100 U/mL penicillin/streptomycin. The pellet mixture was centrifuged at 500× *g* for 5 min at room temperature to obtain a clean cell pellet. This process was done three times to ensure the complete removal of blood cells from the granulosa cell pellet. RNALater (Invitrogen, AM7021, Waltham, MA, USA) was added to the clean pellet of granulosa cells, which was stored at −80 °C until further processing.

### 2.3. RNA Isolation, Transcriptomics, and Metabolomics Data Generation

Total RNA was extracted from granulosa cells collected at the time of harvest from eight fertile and five subfertile heifers (*n* = 13) using a standard protocol for Trizol reagent (Ambion Life Technologies, 15596018, Carlsbad, CA, USA), as we previously described [19]. The Zymo Research RNA Clean and Concentrator Kit (Zymo Research, R1017, Irvine, CA, USA) was used to purify the RNA and digest any gDNA. The concentration and RNA integrity number (RIN) of each sample were then evaluated using the Agilent Bioanalyzer RNA 6000 Nano Kit (Agilent, 5067-1511, Santa Clara, CA, USA) according to the manufacturer’s protocol. The samples with a RIN > 6 were subjected to library preparation and sequencing at Discovery Life Sciences using the NovaSeq platform (Hudson Alpha Institute of Biotechnology, Huntsville, AL, USA), and paired-end 100 bp reads were generated for each sample.

The follicular fluid collected from the ovaries of 13 heifers was subjected to GCTOF-MS by West Coast Metabolomics Center (WCMC, UC Davis, CA, USA). In brief, the analyses were performed using an Agilent 6890 GC fitted with a Gerstel automated liner exchange system (ALEX; Gerstel, Mülheim, Germany) and a Gerstel cold injection system (CIS). The injection volume was 0.5 µL with an injection speed of 10 µL/s. Chromatographic separation was achieved on a Rtx-5Sil MS capillary column (30 m × 0.25 mm internal diameter, 0.25 µm film thickness; 95% dimethyl/5% diphenylpolysiloxane) supplied by Restek Corporation. Helium was used as the carrier gas at a constant flow rate of 1 mL/min. The oven temperature program began at 50 °C (held for 1 min), then ramped at 20 °C/min to 330 °C, with a final hold of 5 min. Mass spectral data were acquired using a Leco Pegasus IV mass spectrometer operating at unit mass resolution. Spectra were collected at 17 scans per second over a mass range of 80–500 Da with an ionization energy of −70 eV. Raw data files were processed using ChromaTOF (v2.32). Absolute spectral intensities were further refined with filtering algorithms implemented in BinBase, a metabolomics database platform. Quantification was based on peak height, using the unique ion as the default metric rather than peak area. The resulting binned dataset was subsequently normalized and scaled to minimize bias introduced by sample preparation and instrumental variability.

### 2.4. Differential Expression and Metabolite Abundance Analysis

Raw RNA sequencing data from the granulosa cells were quality-checked using FastQC v0.11.9 and MultiQC v1.12. The quality was determined based on average read length, adapter content, over-represented sequences, and Phred sequence quality scores [20]. Reads were mapped to Ensembl’s ARS UCD1.3 *Bos taurus* genome reference (https://useast.ensembl.org/Bos_taurus/Info/Index, release 111, accessed on 31 July 2025) through the STAR aligner v2.7.5 [21]. The -quantMode geneCounts flag in STAR was used to obtain raw gene counts [21]. Raw counts per gene were transformed using edgeR v3.28.1 to counts per million (CPM) [22]. MultiQC v1.12 was used for post-mapping quality control [20]. Only genes with CPM > 1 in a minimum of 5 samples (the size of the smallest group) were retained. To test for potential biases between groups, birth weight, weaning weight, and RTS, as well as heifer source and harvest group, were evaluated through principal component analysis and ANOVA. DESeq2 v1.26.0 was used to identify differentially expressed genes (DEGs) [23]. Heifer classification (fertile or subfertile), and harvest groups were included in the negative binomial GLM model implemented into DESeq2. DEGs were considered significant at *p*-value ≤ 0.05 and an absolute log2 fold change > 0.5. The up- or downregulation of DEGs was based on the direction of the log2 fold change in subfertile heifers.

Raw metabolite data files included peak intensities for the quantification ion (*m*/*z* value) at the specified retention index. The data were analyzed using Metaboanalyst 6.0. For each group of metabolites, only those with a relative standard deviation ≤ 0.10 were used based on the raw counts. The raw data were log-normalized and subjected to t-tests. Differentially abundant metabolites (DAMs) were taken as significant when *p*-value  <  0.05. These approaches, rather than more stringent methods, were used because our objective was to prioritize genes and metabolites for downstream integration and co-expression analyses. We aimed to retain biologically relevant candidates to help identify coordinated molecular patterns across regulatory layers. This strategy allows identification of features that show both statistical support and biologically meaningful magnitude of change, while remaining appropriate for an exploratory multi-omics framework. Because the number of biological replicates was limited (8 fertile and 5 subfertile animals), the statistical power to detect subtle expression differences was reduced. Thus, identified candidate genes should be validated in independent populations with larger sample sizes.

### 2.5. Gene and Metabolite Co-Expression Networks

The expression patterns and relationships between the regulatory layers (metabolome and transcriptome) were determined using partial correlation and information theory (PCIT) [24]. To this end, 13,887 genes were normalized using CPM for the gene networks (see Section 2.4), while 189 normalized annotated metabolites were used for the metabolite network. Separate networks were constructed for the fertile and subfertile groups. The significant pairs were selected based on partial correlation values greater than absolute r ≥ 0.95 for genes and r > 0.80 for metabolites (*p*-value < 0.05). The stringent correlation thresholds were selected to minimize spurious associations and retain only the strongest and most biologically meaningful relationships. This also reduces the likelihood of false-positive connections in network construction when statistical power is limited by sample size. Additionally, significant pairs were filtered based on the identified DEG or DAM. Network topology measures, such as degree and betweenness centrality, were calculated using the Network Analyzer tool in Cytoscape v3.8.2 to identify hub nodes for genes and metabolites [25,26]. Genes and metabolites with a mean + 2SD (standard deviation) of degree measure were classified as “hub targets.” In network theory, nodes with extensive connectivity (high-degree genes or metabolites) maintain central positions in the network and are often considered key regulators of important biological pathways.

To examine changes in node connectivity and edge rewiring between the fertile and subfertile groups, we employed the Cytoscape plug-in DyNet [27]. DyNet builds a reference network from co-expressed pairs and pinpoints the nodes with the greatest structural rewiring across groups. Differential connectivity analysis was then performed to identify genes or metabolites with altered interaction patterns. To this end, we computed the differential connectivity (DK) index, defined as the difference in connectivity between a node (gene or metabolite) in fertile and subfertile networks. The DK values were standardized to z-scores (z−score=DKi−μ/σ), where μ and σ represent the mean and standard deviation of DK across nodes, respectively. Nodes with z-scores outside ±1.96 (*p*-value ≤ 0.05) were considered significantly differentially connected and were termed “differentially connected targets”, as we previously described [28]. All networks were generated and visualized in Cytoscape v3.8.2.

### 2.6. Gene–Metabolite Integration and Pathway Over-Representation Analysis

To shed light on the complex relationship between genes and metabolites within the fertile and subfertile groups, we created a single co-expression network combining normalized gene (13,887) and metabolite (189) data using PCIT. Only gene–metabolite pairs with absolute correlation values ≥ 0.95 (*p*-value < 0.05) and co-expressed with a hub or differentially connected targets (genes and metabolites) (unique targets) were considered for further analysis. To gain insight into the specific networks involved in fertility status, the correlated pairs were filtered based on the “unique” targets. The networks with significant gene–metabolite pairs for the fertile and subfertile groups were visualized in Cytoscape. Likewise, node and edge rewiring within the interaction networks was performed using DyNet (Cytoscape v3.8.2), and the degree connectivity of each gene and metabolite was calculated using Network Analyzer. For the significant gene–metabolite pairs, joint-pathway analysis was performed using MetaboAnalyst 6.0, with *Bos taurus* species set as the reference. We used a joint pathway analysis with a hypergeometric test for enrichment, degree centrality as a topological measure, and combined pathway-level queries for integration. Pathways with *p*-value ≤ 0.05 were considered significant.

### 2.7. Validation of Top Targets with RT-qPCR

The significantly up- or downregulated genes, also identified as hub targets, were validated in a set of 8 granulosa RNA samples (4 per group) collected from non-pregnant heifers following the breeding season. Total RNA was extracted using the Trizol protocol, as described in Section 2.3. The RNA concentration was measured using a Qubit RNA Broad Range Assay Kit (Thermo Fisher Scientific, USA) on a Qubit 3.0 Fluorometer. The total RNA was reverse transcribed using qScript cDNA Supermix (Quanta Biosciences Inc., Beverly, MA, USA). The complementary DNA (cDNA) was diluted 1:5 (*v*:*v*). For specificity testing, cDNA was used for qPCR (quantitative polymerase chain reaction) with AccuStart II PCR SuperMix (QuantaBio, Beverly, MA, USA). qPCR products were visualized on a 1% agarose gel. Efficiency testing was performed using qPCR (PerfeCTa SYBR Green FastMix, QuantaBio, Beverly, MA, USA) of a four-fold serial dilution, including six concentrations of cDNA generated from random samples. The efficiency of each primer was calculated using the Pfaffl method [29]. The final reaction consisted of 2 µL diluted cDNA, 100 nM of each primer, and PerfeCTa SYBR Green Supermix (Quanta Biosciences Inc., Beverly, MA, USA) to a total reaction volume of 10 µL. The primer sequences used for RT-qPCR are given in Supplementary Table S13. The reactions were assayed on the Roche LightCycler 480. The fold change in mRNA expression was calculated using the ΔΔ^Ct^ method [30], normalizing the cycle threshold (Ct) of the target gene to the housekeeping gene *H2A* on each plate. The statistical analysis and graphs were constructed using GraphPad Prism v6.01 (GraphPad, San Diego, CA, USA), and the results are reported as mean ± SD. Statistical analysis was performed using the Mann–Whitney test (non-parametric), and a *p*-value < 0.05 was considered statistically significant.

## 3. Results

### 3.1. Transcriptome Profiling from Granulosa and Differential Gene Expression

RNA-Seq expression profiles of granulosa cells from fertile (*n* = 8) and subfertile (*n* = 5) heifers were used to explore biological pathways underlying fertility status. Sequencing across all samples yielded an average of 27 million reads per sample, of which 91.72% were uniquely mapped to the reference genome (Supplementary Table S1). After quality control and removal of lowly expressed genes, 13,887 genes out of 36,075 in 13 samples remained for further analysis.

**Figure 1:**
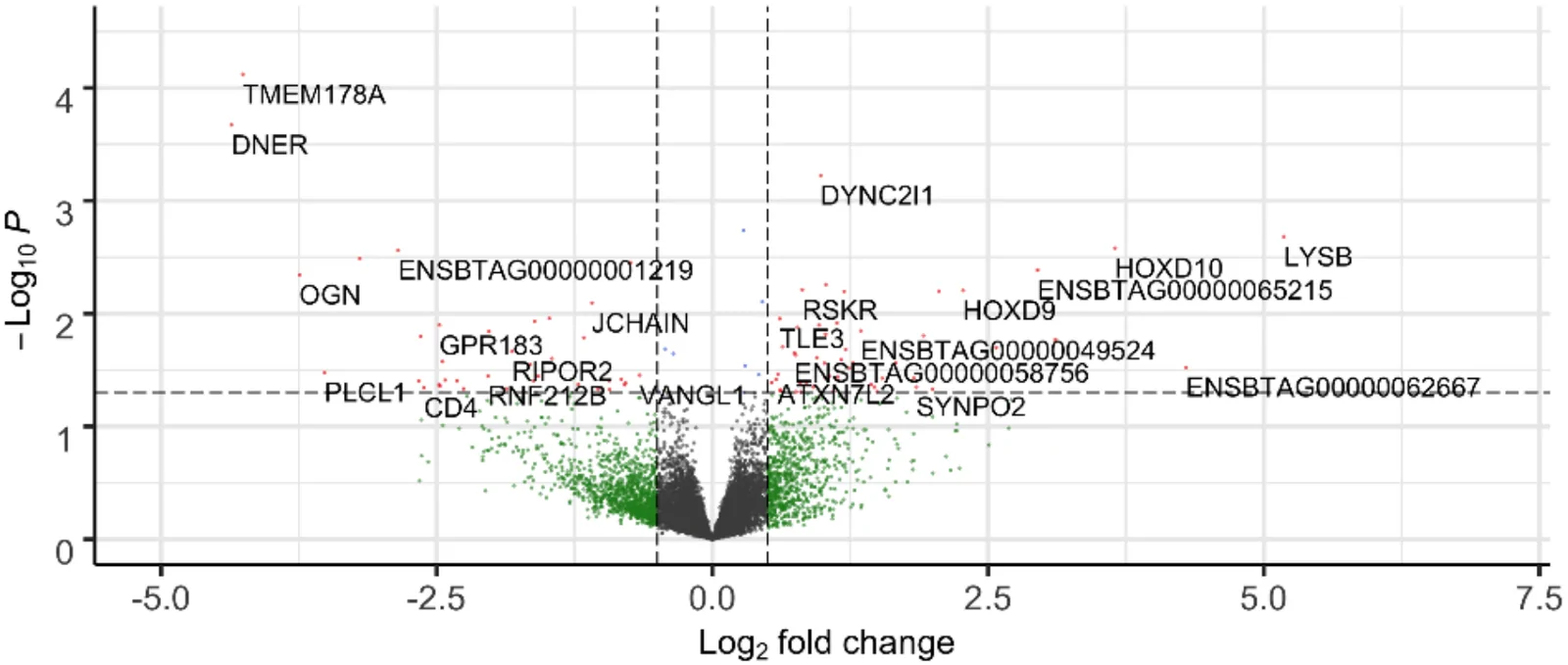
Differentially expressed genes between the fertile and subfertile groups are represented in the volcano plot. Each dot represents a gene. Gene expression levels are represented by log2fold change (x-axis) and –log10 *p*-value (y-axis). Gene significance is color-coded as grey (non-significant genes that did not cross the threshold of *p*-value or fold-change); green (genes with absolute (log2 fold change ≥ 0.5)); blue (genes with a significant *p*-value); and red (90 DEGs with *p*-value ≤ 0.05 and absolute (log2 fold change ≥ 0.5)). The genes were classified as up- or downregulated based on the sign of the log2 fold change in the subfertile group.

We identified 90 significantly differentially expressed genes (Figure 1, Supplementary Table S2) between the fertile and subfertile groups (*p*-value < 0.05; absolute log2fold change > 0.5), including 62 protein-coding, 10 lncRNAs, 1 pseudogene, 2 rRNAs, and some novel genes. Among the 90 DEGs, 40 were downregulated, and 50 were upregulated in the subfertile group. The significantly upregulated genes included *CXCL12*, *DYNC211*, *LYSB*, transcription factors from the *HOX* family (*HOXD9*, *HOXD10*, *HOXD11*), protocadherin genes (*PCDH8* and *PCDH20*), while downregulated genes included *CALCRL*, *DNER*, *GADD45G*, *PLCE1*, and genes from the cluster of differentiation family (*CD4*, *CD52*, and *CD247*).

### 3.2. Differential Gene Network Analysis

**Figure 2:**
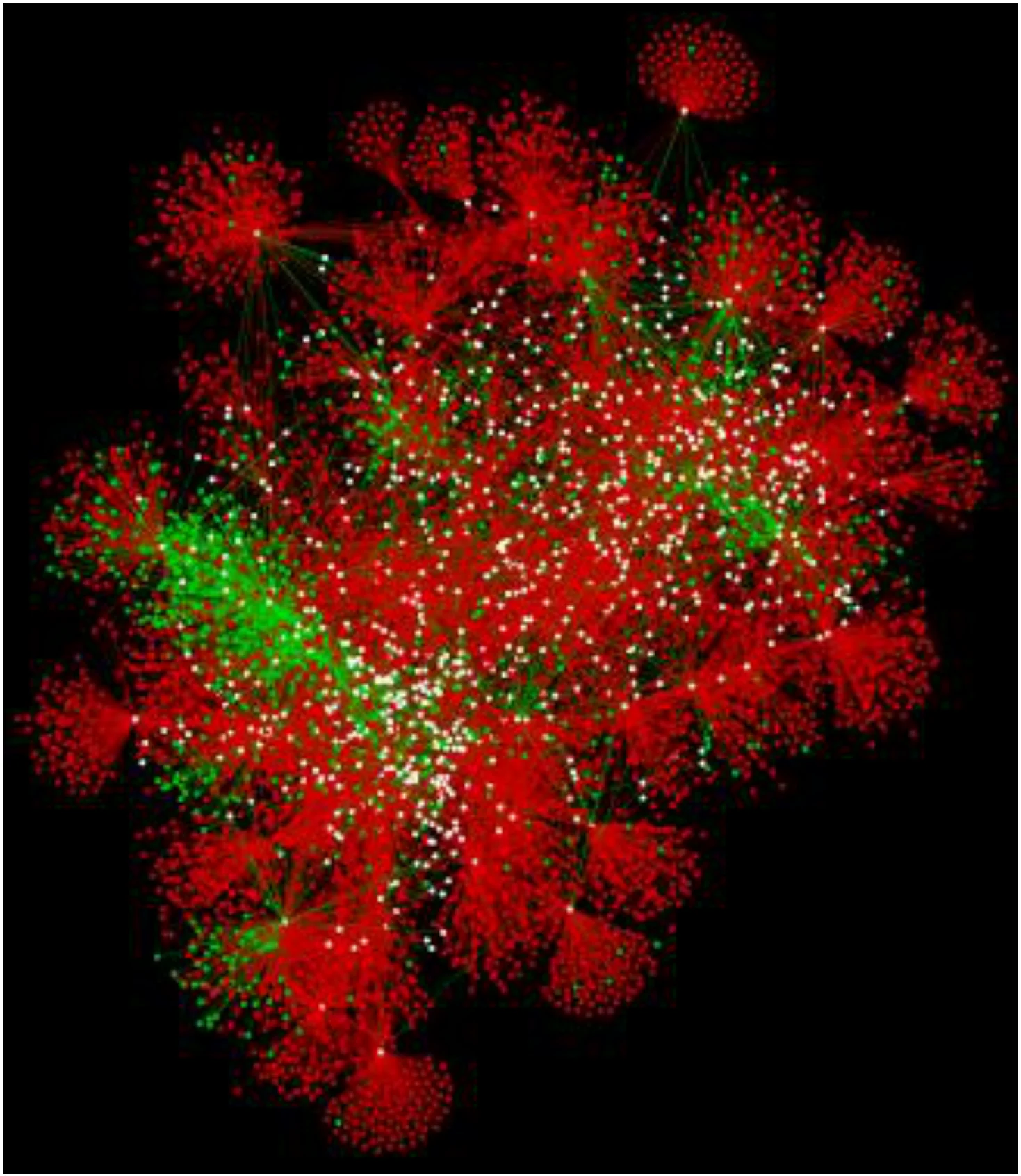
Central reference network of co-expressed genes between fertile and subfertile groups. The network was constructed using DyNet and visualized on Cytoscape. The network comprises 7244 nodes (genes) and 13,048 edges (interactions). The unique nodes are green (fertile group) and red (subfertile group). The white nodes represent the genes shared between fertile and subfertile networks.

Using the PCIT approach, we identified 4,454,369 and 1,149,465 significantly correlated pairs in the fertile and subfertile groups, respectively. To retrieve biological information and reduce data dimensionality, the correlated pairs were filtered to include only those that include one of the 90 identified significant DEGs and an absolute correlation greater than 0.95. After filtering, 2167 and 10,926 gene pairs were maintained (Supplementary Table S3), corresponding to 1644 and 6389 nodes (Supplementary Table S4) for the fertile and subfertile groups, respectively. The hub genes were identified using the average + 2SD of the degree measure calculated through the network analyzer in Cytoscape. We identified 16 and 87 hub targets in the fertile and subfertile networks (Supplementary Table S4). Furthermore, in the subfertile group, 71 out of 87 genes were exclusive hub targets, including *PTCD2*, *GADD45G*, *CALCRL*, *DNER*, *ZPBP2*, *JCHAIN*, *PCDH8*, *CPEB1*, and *PLCE1*. We used DyNet to construct a central reference network for both groups using 7244 nodes (genes) and 13,048 edges (interactions) (Figure 2, Supplementary Table S5). The differential connectivity between the groups was calculated using the frequency of gene degree for 7244 genes. We identified 84 differentially connected genes, out of which 82 gained connectivity in the subfertile network. The remaining 2 genes, *CEMIP* and *GPR183*, were less connected in the subfertile network.

### 3.3. Differential Metabolite Abundance and Network Analyses

**Figure 3:**
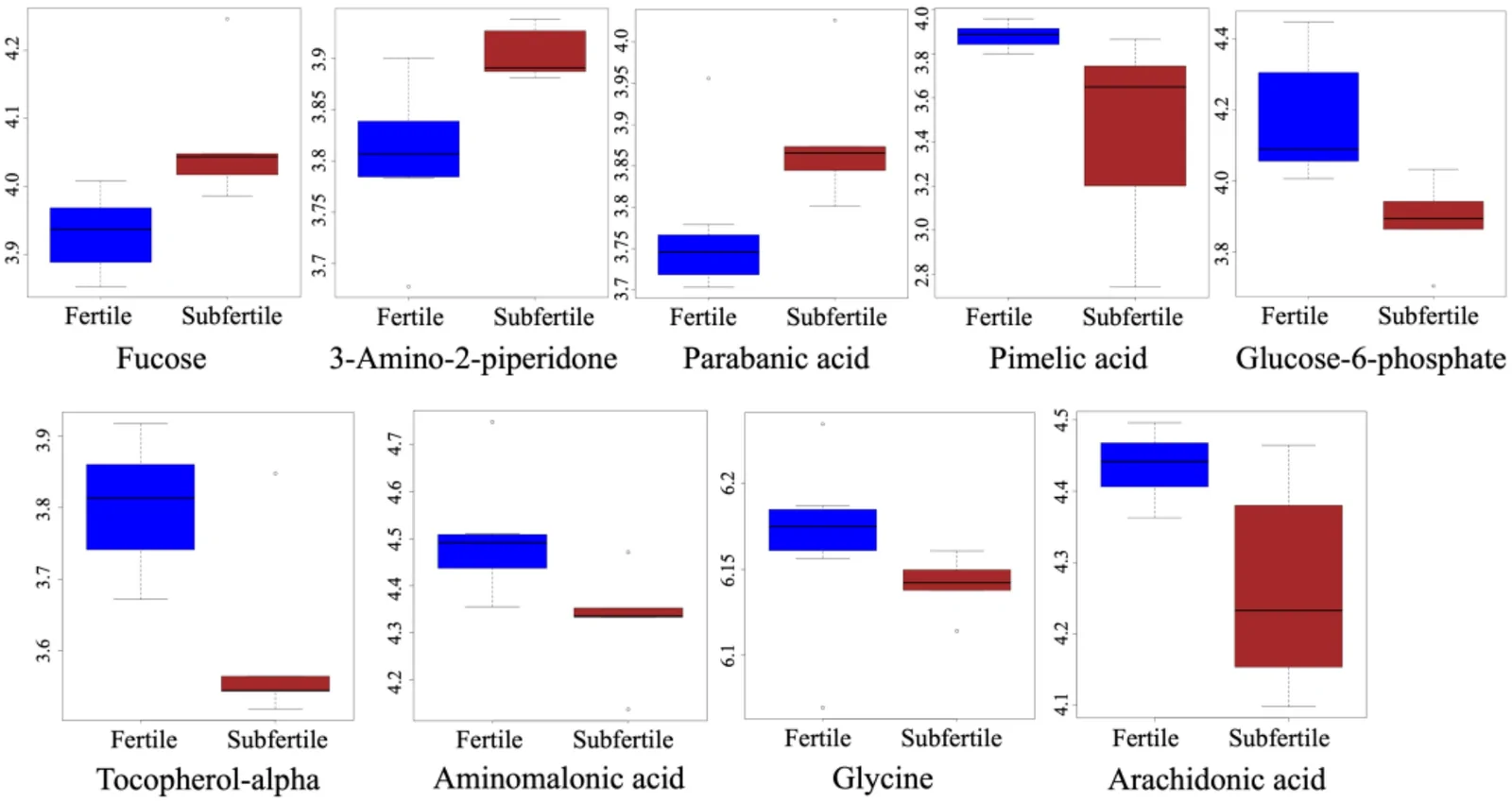
Levels of differentially abundant metabolites in fertile (blue) vs. subfertile (brown) groups. The x-axis represents the groups, and the y-axis represents the normalized metabolite levels.

Untargeted metabolomics of follicular fluid from fertile and subfertile heifers was used to explore biological pathways that might affect pregnancy outcomes as a measure of fertility. After normalization and filtering, 189 annotated metabolites were analyzed. The Wilcoxon rank-sum nonparametric test identified nine differentially abundant metabolites (DAMs; *p*-value < 0.05). The metabolites included fucose, 3-amino-2-piperidone, and parabanic acid at higher levels in the subfertile group, while pimelic acid, glucose-6-phosphate, tocopherol alpha, aminomalonic acid, glycine, and arachidonic acid are at lower levels in the subfertile group (Figure 3, Supplementary Table S6).

**Figure 4:**
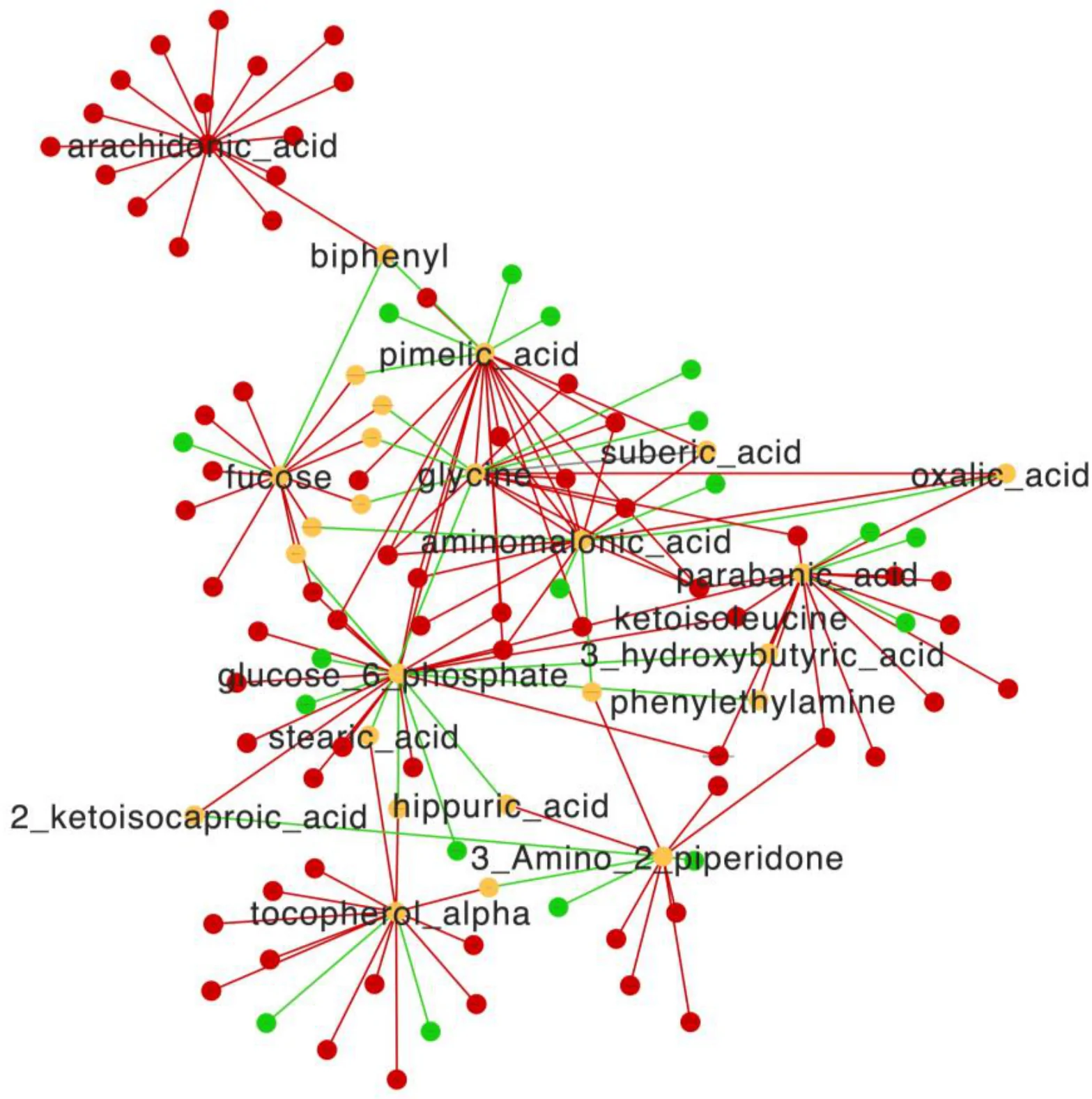
Central reference network of metabolites between fertile and subfertile groups. The network was constructed using DyNet and visualized on Cytoscape. The network comprises 111 nodes (metabolites) and 151 edges (interactions). The unique nodes are green (fertile group) and red (subfertile group). The yellow nodes represent the genes shared between fertile and subfertile networks.

The 189 annotated metabolites were subjected to the PCIT approach for the metabolite network. We identified 2202 and 1875 significantly correlated pairs in the fertile and subfertile groups, respectively. To retrieve biological information and reduce data dimensionality, the correlated pairs were filtered using the nine significant DAMs and an absolute correlation threshold of 0.8 (*p*-value < 0.05). After filtering, 36 and 116 metabolites were maintained (Supplementary Table S7), corresponding to 43 and 93 nodes (Supplementary Table S8) for the fertile and subfertile groups, respectively. The hub metabolites were identified using a network analyzer based on the average + 2SD of the degree measure. Four and eight hub metabolites were identified in the fertile and subfertile network (Supplementary Table S8). Arachidonic acid, parabanic acid, tocopherol alpha, and fucose were exclusive hub targets to the subfertile network. We used DyNet to construct a central reference network for the fertile and subfertile group using 111 nodes (metabolites) and 151 edges (interactions) (Figure 4, Supplementary Table S9). The differential connectivity between the groups was calculated using the frequency of metabolite degrees for 111 metabolites. After calculating z-scores, we identified five differentially connected metabolites, including arachidonic acid, parabanic acid, tocopherol alpha, fucose, and pimelic acid. All the metabolites gained connectivity in the subfertile network.

### 3.4. Gene–Metabolite Interaction Network and Pathways

**Figure 5:**
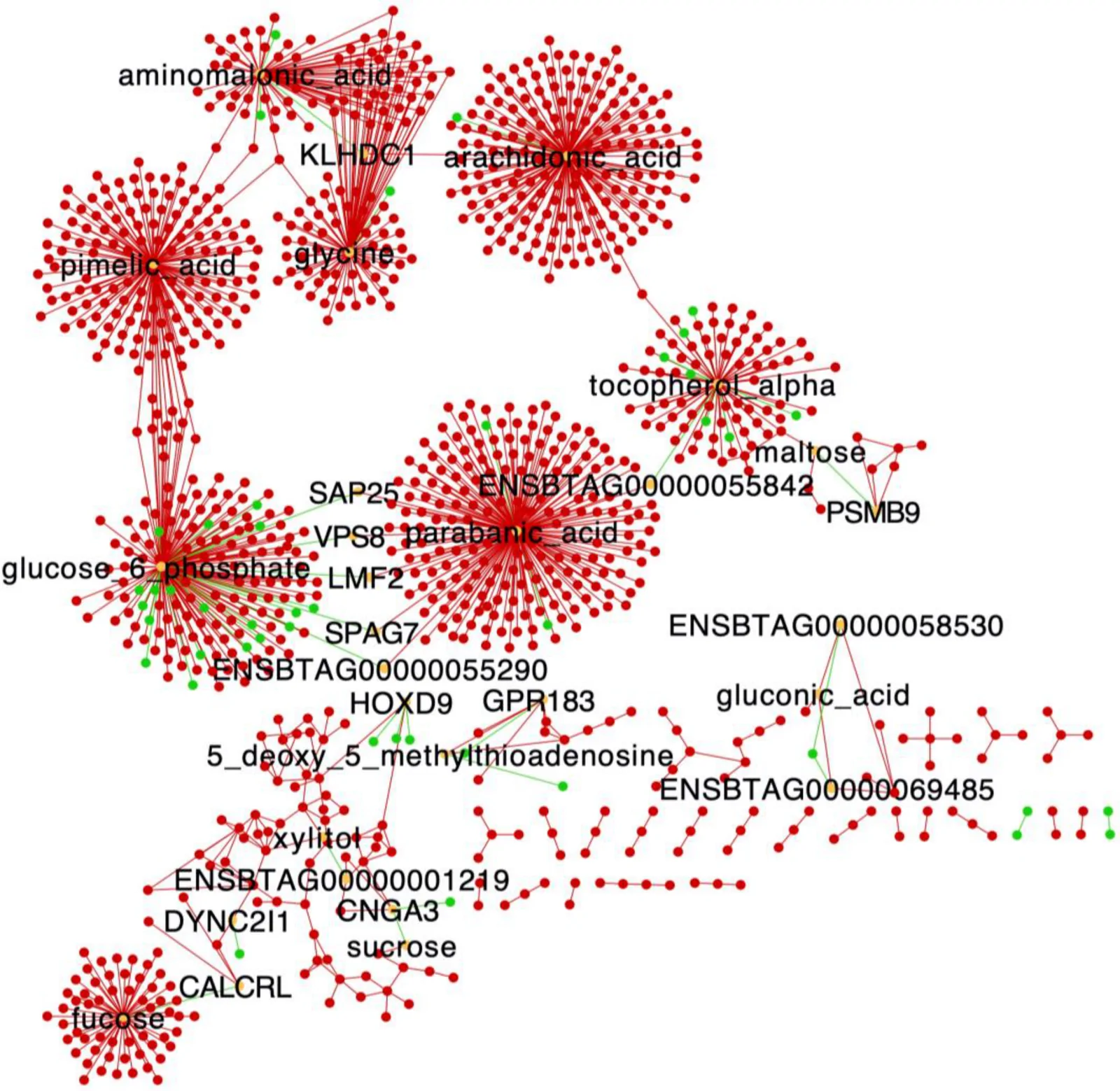
Central reference network of significant gene–metabolite pairs between fertile and subfertile groups. The network was constructed using DyNet and visualized on Cytoscape. The network comprises 1096 nodes (gene–metabolite pairs) and 1155 edges (interactions). The unique nodes are green (fertile group) and red (subfertile group). The yellow nodes represent the genes shared between fertile and subfertile networks (Supplementary Table S11).

To identify significant co-expressed pairs, including gene-gene, metabolite-metabolite, and gene–metabolite pairs, we performed a combined PCIT analysis for 189 metabolites and 13,887 genes. The combined PCIT resulted in 4,513,611 and 1,167,732 in the fertile and subfertile groups, respectively. Only significant gene–metabolite correlations were filtered based on unique targets in the fertile and subfertile groups. To identify those targets, we considered the hub genes and metabolites (Supplementary Tables S4 and S8) and the significantly differentially connected genes and metabolites (Supplementary Tables S5 and S9) for each group. After filtering, we retrieved 55 and 1101 gene–metabolite pairs from the fertile and subfertile network groups, respectively (Supplementary Table S10). Interestingly, parabanic acid, arachidonic acid, glucose 6-phosphate, pimelic acid, glycine, alpha-tocopherol, aminomalonic acid, and fucose had a very high degree of connectivity (degree > 50), representing a higher number of interactions with the genes and metabolites in the subfertile group (Figure 5, Supplementary Table S11). Furthermore, pimelic acid was exclusively correlated with the genes and metabolites only in the subfertile group.

These pairs were then used for functional over-representation analysis in MetaboAnalyst (Supplementary Table S12). In the fertile group, the gene–metabolite pairs were significantly over-represented (*p*-value < 0.05) in Starch and Sucrose Metabolism, Carbohydrate digestion and absorption, Pentose and Glucuronate interconversions, Efferocytosis, Glycine, serine, and threonine metabolism, and Purine metabolism. In contrast, Aminoacyl-tRNA biosynthesis, Alanine, aspartate and glutamate metabolism, Oxidative phosphorylation, Arginine biosynthesis, and Hippo-signaling pathway were significantly over-represented in the subfertile group (*p*-value < 0.05). In addition to these pathways exclusive to each group, ABC transporters, Ferroptosis, Mineral absorption, and Glyoxylate and Dicarboxylate metabolism were common significant pathways in both the fertile and subfertile groups.

### 3.5. mRNA Expression of the Identified DEGs and Hub Targets

**Figure 6:**
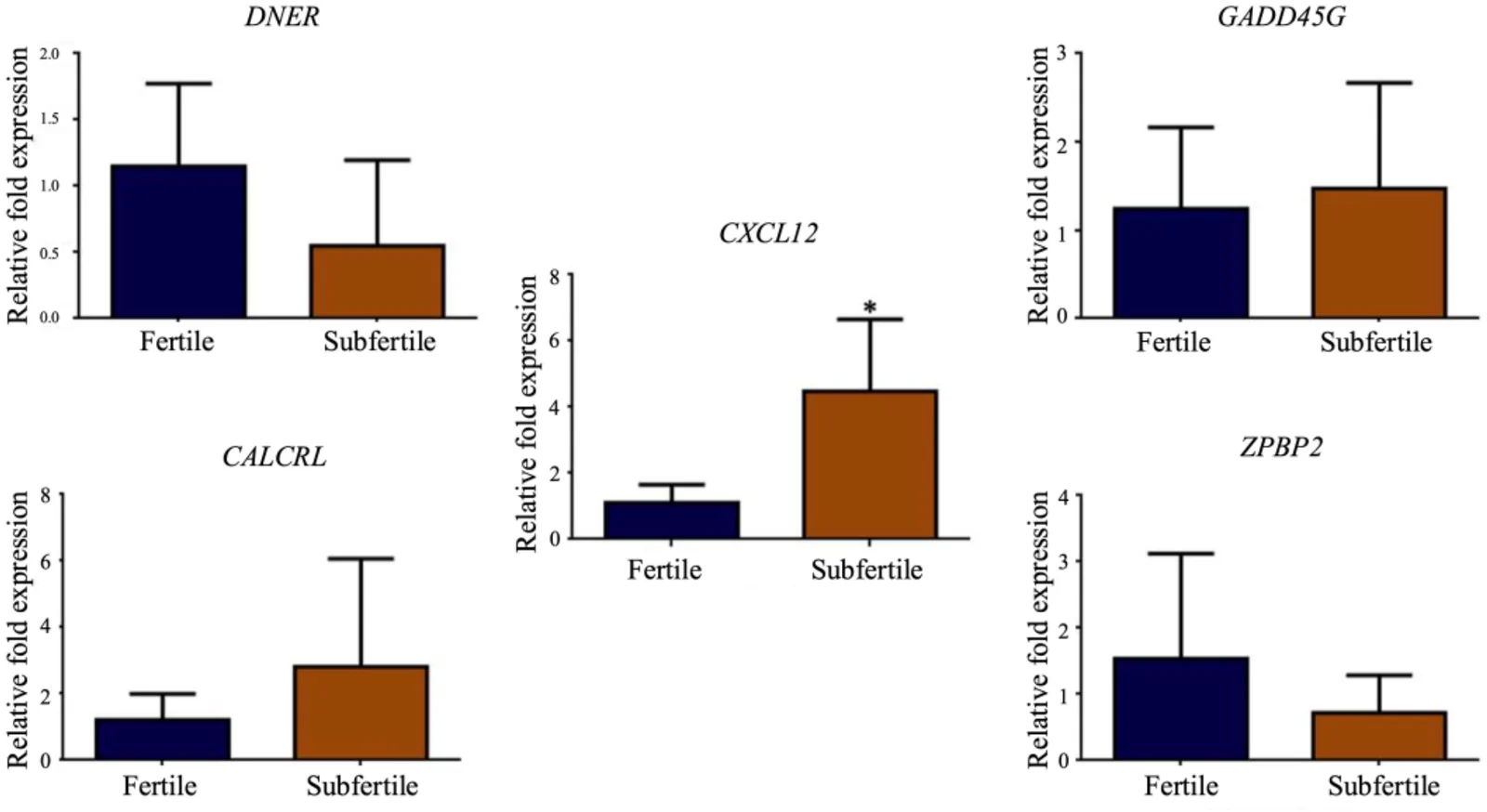
Comparison of relative transcript levels between fertile (blue) and subfertile (brown) heifers. Data presented as Mean + SD. * Significant at *p*-value < 0.05.

Based on the results from DEGs and hub targets, five genes (*DNER*, *ZBPB2*, *CXCL12*, *CALCRL*, and *GADD45G*) were selected to quantify their expression with qPCR (Primer list in Supplementary Table S13). Consistent with the results mentioned above, *CXCL12* was significantly upregulated (*p* = 0.009) in the subfertile heifers (Figure 6). *ZBPB2* and *DNER* were identified as having low expression in subfertile heifers (same trend as observed in the RNA-Seq approach); however, the results were not significant (*p*-value > 0.05).

## 4. Discussion

In cattle production, the selection of replacement heifers has profound implications for long-term reproductive success and herd longevity [31,32]. While producers traditionally rely on phenotypic indicators related to reproductive maturity to reduce the likelihood of retaining reproductively inefficient heifers, subfertility remains a persistent challenge, particularly in heifers that fail to conceive following AI and subsequent natural service.

Herein, we employed an integrated gene–metabolite approach, combining granulosa cell transcriptomic analysis and follicular fluid metabolomic analysis to identify gene and metabolite signatures associated with fertility status. Using differentially expressed genes (DEGs) and differentially abundant metabolites (DAMs), co-expression network analyses were constructed to identify key hubs, differentially connected genes and metabolites, and biologically relevant gene–metabolite interactions underlying subfertility. We identified 90 DEGs from the granulosa cells and 9 DAMs from the follicular fluid between the fertile and subfertile groups. Among the DEGs identified, *ENSBTAG00000035855*, *DNER*, *ENSBTAG00000066429*, *S100A12*, *ENSBTAG00000058401*, and *UBD* were significantly downregulated, while *NETO2* was significantly upregulated in subfertile heifers. Interestingly, these genes were identified as differentially expressed in our previous study of the endometrium [33].

For the network analysis, increased gene connectivity observed in subfertile heifers compared with the fertile group suggests network rewiring of major regulatory pathways in response to fertility status, providing novel insights into the molecular regulation of reproductive success in beef heifers. At the biological level, ovarian function and follicular dynamics play central roles in determining fertility outcomes. Granulosa cells within the ovarian follicle regulate highly coordinated processes essential for follicular development and oocyte competence, while follicular fluid derived from plasma transudate and secretions from granulosa cells and the oocyte serves as a critical microenvironment for oocyte growth and maturation [34]. Despite their importance, limited information is available on the cellular and molecular mechanisms in granulosa cells and follicular fluid that differentiate fertile from subfertile beef heifers.

In our study, *DNER* was identified as a hub gene and showed a significant correlation (r > 0.95) with dodecanol in the subfertile group. The *DNER* (Delta/Notch-like EGF-related receptor) gene encodes a transmembrane protein that binds Notch1 [35]. Notch1 is linked to early pregnancy development through cell survival, growth, implantation, and placentation, in mice and humans [36]. Notch 1 protein expression is higher in the endometrium of fertile women compared to infertile women [37]. Similarly, low *DNER* methylation levels were observed in early chorionic tissue and have been linked to the occurrence and development of ectopic pregnancies in humans [38]. Dodecanol, a 12-carbon fatty alcohol, serves as a specific indicator of ovulation, with a sharp increase in its concentration in human saliva at that time [39]. Although we found a strong correlation between DNER and dodecanol, we did not find dodecanol as differentially abundant or a hub metabolite in our study.

Among the DEGs identified, *CD4*, *CD247*, *CD52*, *GIMAP6*, *GPR183*, *JCHAIN*, *TAP1*, *PSMB9*, *UBD*, *TBC1D10C*, *RIPOR2*, and *S100A12* are involved with immune response. Interestingly, all these genes were downregulated in the subfertile heifers. Proper immune modulation is critical for fertility, as ovulation and implantation/attachment are inflammatory-like processes that require tightly controlled immune responses [40]. Dysregulation of immune pathways may lead to excessive inflammation or inadequate immune tolerance, impairing follicular development, luteal function, or embryo attachment in subfertile heifers. In contrast, *CXCL12* was significantly upregulated (in both RNA-Seq and qPCR) and showed a connectivity gain in the subfertile group. Interestingly, *CXCL12* was correlated with gluconic acid in the subfertile group. *CXCL12*, also referred to as stromal cell–derived factor-1 (SDF-1), is a small homeostatic chemokine belonging to the CXC subfamily [41]. *CXCL12* and its receptors, including *CXCR4* and *CXCR7*, are widely expressed in placental tissue and play essential roles in key reproductive processes including implantation/attachment, placentation, and embryogenesis in humans and cattle [42,43]. Furthermore, expression of the CXCL gene family in the endometrium was negatively associated with pregnancy/AI in Brahman cows [44] and associated with inflammatory response in crossbred cows [45]. Additionally, the CXCL12-CXCL4 signaling axis contributes to maternal–fetal immune tolerance and vascular remodeling during early pregnancy [42]. Given its significant role in early gestation and attachment, one possible explanation for the observed upregulation of *CXCL12* in granulosa cells from subfertile heifers is an adaptive or stress-related response to a dysregulated follicular microenvironment rather than an enhancement of reproductive competence. CXCL12–CXCR4 signaling has been implicated in granulosa cell survival, immune cell recruitment, and follicular remodeling in mammalian ovaries, where *CXCL12* production by luteinizing granulosa cells contributes to reduced apoptosis and increased cell survival in association with local immune interactions in preovulatory follicles, suggesting its role in maintaining cell viability under suboptimal conditions [46]. In the context of impaired folliculogenesis or endocrine imbalance, altered hormonal signaling can upregulate chemokine expression, including *CXCL12* [47]. Collectively, these findings suggest that increased *CXCL12* expression in subfertile heifer granulosa cells may also be a marker of follicular stress, defective angiogenic signaling, or a disrupted gonadotropin response. Additionally, *CXCL12* was over-represented for the axon guidance pathway in the subfertile network. Both excessive and insufficient expression and activation of CXCL12/CXCL4 signaling disturb neuronal migration [48] and axon guidance [49]. Maternal immune activation caused by an infection during pregnancy can alter the expression of the genes that are involved in the regulation of axon guidance in the fetus. Impaired axon guidance is closely linked to disrupted nervous system development, which can lead to low embryo development, structural defects, and embryonic lethality [50].

We identified nine differentially abundant metabolites, out of which fucose, tocopherol-alpha, aminomalonic acid, glycine, and arachidonic acid were found at lower levels in subfertile heifers. Although these metabolites were at lower levels, they were differentially associated with increased network connectivity in the subfertile group. Tocopherol-alpha (Alpha-tocopherol), the most active form of Vitamin E, with low levels in cows being directly linked to reproductive failures, including increased pregnancy loss, embryonic death, abortions, and retained fetal membranes (RFM) [51]. Alpha-tocopherol supplementation in periparturient dairy cows was shown to improve fertility [52]. Additionally, in vitro, alpha-tocopherol increased cleavage and blastocyst formation due to its antioxidant properties and reduction of reactive oxygen species (ROS) [53]. Antioxidants are crucial for proper female reproductive function, as they participate in oocyte metabolism, endometrial maturation via activation of antioxidant signaling pathways, and hormonal regulation [54]. Low levels of alpha-tocopherol could be attributed as one of the reasons for subfertility in heifers. Interestingly, alpha tocopherol was correlated with 70 genes in the subfertile network. One of the genes was *TAP1*, which was differentially expressed and a hub gene in the subfertile group. The *TAP1* gene belongs to a family of transporters associated with the antigen processing complex (TAP) and, along with *TAP2*, is crucial for immune peptide processing and MHC class I assembly and is linked to female fertility through its role in immune regulation at the maternal-fetal interface [55]. Expression quantitative trait loci (eQTL) in these genes affect implantation success, time to pregnancy, and pregnancy loss, with specific mutations potentially causing unexplained infertility in humans [55].

Immune-related pathways identified in this study may represent potential targets for improving reproductive resilience; however, strategies to modulate immune function in cattle require careful consideration of the balance between protective immunity and excessive inflammation. Potential approaches include nutritional interventions that support immune homeostasis, such as optimizing antioxidant status and providing bioactive compounds that influence inflammatory pathways, as well as management practices aimed at reducing physiological stressors that negatively impact immune function and fertility. Additionally, targeted regulation of immune signaling pathways through improved understanding of key immune-related genes and metabolites may provide opportunities for enhancing the ovarian microenvironment and supporting oocyte competence. However, the application of immune-modulating strategies to improve fertility remains an emerging area of research, and further studies are needed to determine whether modulation of these pathways can causally improve reproductive outcomes in beef cattle.

Among other significant gene–metabolite pairs, we identified arachidonic acid as differentially abundant with low abundance in the subfertile heifers. Arachidonic acid was identified as a hub metabolite; in the fertile group, it correlated with only 1 gene (RIOK2), whereas in the subfertile group, it correlated with 169 genes. Among these 169 genes, arachidonic acid was significantly correlated with *ENSBTAG00000058249*, *MYCBP*, *PACSIN3*, and *PCSK1N* in the subfertile group. These genes were differentially expressed in the endometrium of beef heifers, with *ENSBTAG00000058249* (lncRNA), *PACSIN3*, and *PCSK1N* significantly upregulated and *MYCBP* significantly downregulated in the subfertile group [33]. Arachidonic acid (AA), an omega-6 polyunsaturated fatty acid, plays a critical role in bovine female fertility, primarily by acting as a precursor to prostaglandins (PGs) necessary for ovulation, luteal function, and uterine health [56]. In a mouse study, arachidonic acid supplementation improved the ovarian reserve, increased the oocyte number and quality, and restored fertility [57]. The low level of arachidonic acid and gain of network connections in the subfertile group could be attributed to one of the potential candidates to explore for subfertility in heifers.

*CALCRL* (Calcitonin Receptor-Like Receptor) was identified as differentially expressed and downregulated in the subfertile group. It was also identified as a differentially connected hub gene with significant correlations with trans-4-hydroxyproline, saccharic acid, and cellobiose in the subfertile group. *CALCRL* is a G protein-coupled receptor (GPCR) that functions as a key receptor for peptide hormones, specifically adrenomedullin (ADM) and calcitonin gene-related peptide (CGRP) [58]. It forms heterodimeric complexes with receptor activity-modifying proteins (RAMPs 1–3) to determine ligand specificity and regulate physiological processes such as blood pressure, inflammation, and cellular proliferation [58]. In the human endometrium, low expression of calcitonin receptor-like receptor was associated with infertility [59].

We identified Aminoacyl-tRNA biosynthesis; Alanine, aspartate and glutamate metabolism; Oxidative phosphorylation; Arginine biosynthesis; and the Hippo-signaling pathway as significantly over-represented by the gene–metabolite pair in the subfertile group. In humans, while disruption of hippo-signaling disrupts ovarian follicle activation in infertile patients [60], arginine is a multifunctional amino acid required for survival, growth, and development of conceptuses (embryo/fetus and associated extraembryonic membranes) during the peri-implantation period of pregnancy [61]. Oxidative phosphorylation is a critical determinant of fertility in cattle, serving as the primary energy-producing mechanism in oocytes and as an essential component of early embryo development [62]. Similarly, arginine biosynthesis was significantly over-represented in subfertile heifers. Arginine, an essential amino acid, is responsible for the production of various molecules, including nitric oxide (NO) and polyamines, which promote vasodilation and nutrient delivery to the follicle, thereby supporting oocyte growth [63,64].

Although this study provides valuable insight into the molecular mechanisms associated with subfertility in beef heifers, several limitations should be acknowledged. First, the relatively small sample size may limit statistical power through increasing the FDR and the generalizability of the findings. However, similar sample sizes have been used in previous exploratory omics studies in livestock, where the objective is to identify biological features to generate hypotheses for validation in larger cohorts [65,66,67]. Second, samples were collected at a single time point, limiting our ability to capture dynamic changes in gene expression and metabolite abundance throughout the estrous cycle and early pregnancy. Fertility is a temporally regulated process, and longitudinal sampling would provide a more comprehensive understanding of molecular fluctuations associated with successful conception. Our approach utilizing ES-AI followed by natural service was designed to provide all the heifers adequate opportunities to become pregnant. Even with multiple opportunities, it is possible that fertile heifers may still have missed these opportunities. They may not have responded to the ES protocol and have been missed by the bull when they displayed estrus due to the heifers being synchronized. We tried to control this by using two proven sires providing heavy bull power. We also sourced heifers from two different research sites with similar but distinct nutrition programs. Tightly controlling the nutrition throughout the heifers’ development would remove another potential source of variation. Additionally, the gene–metabolite interaction approach identifies associations and network correlations but does not establish causality; therefore, functional validation studies are required to confirm the biological roles of the identified hub genes and metabolites. The qPCR expression trends were consistent with the RNA-Seq results; yet only one of the selected genes was statistically significant. This is because the qPCR analysis provides limited validation of the transcriptomic findings rather than broad experimental confirmation [68]. Therefore, additional validation in larger cohorts is warranted. Finally, although consistency with prior endometrial findings strengthens the biological relevance of some targets, external validation in independent, larger cohorts is necessary before these biomarkers can be reliably applied in fertility prediction or selection programs. The functional interpretation of several candidate genes and pathways is based on evidence from human and murine studies, given the limited information available in bovine models. Although many reproductive processes are evolutionarily conserved across mammals, species-specific differences in ovarian physiology and gene regulation may influence the biological roles of these molecules in cattle. Furthermore, genes, metabolites, and pathways reported were associated with fertility, but we do not establish causal relationships. Consequently, the proposed biological roles of candidate molecules should be interpreted as plausible mechanistic hypotheses considering their known biological functions and previous literature rather than direct evidence of causality. These associations provide a framework for future functional studies to determine whether and how these molecular pathways contribute to fertility regulation in beef heifers. Integrating multi-omics profiles into selection strategies may enhance reproductive efficiency, improve herd longevity, and support more sustainable beef production systems.

## 5. Conclusions

This study provides novel insights into the molecular signatures underlying subfertility in beef heifers by integrating granulosa cell transcriptomics with follicular fluid metabolomics. We reported coordinated gene–metabolite interactions and network rewiring events that distinguish fertile from subfertile animals. The increased connectivity observed in subfertile heifers suggests a systemic reorganization of regulatory pathways, potentially reflecting compensatory or stress-related responses within the follicular microenvironment. Key findings highlight the involvement of immune modulation, oxidative balance, and angiogenic and endocrine signaling in fertility outcomes. Hub genes, along with metabolites, emerged as central components of altered networks in subfertile heifers. Collectively, these findings advance our understanding of the biological mechanisms contributing to subfertility and provide a foundation for the development of predictive biomarkers for heifer fertility.

## Data Availability

All relevant data are included in the paper and its Supplementary Materials. All sequencing data is deposited on the GEO database and will be made publicly available at acceptance of the manuscript.

## Supplementary Materials

The following supporting information can be downloaded at https://www.mdpi.com/article/10.3390/genes17080889/s1, Table S1. Summary and mapping statistics of RNA-Sequencing data from granulosa cells of fertile and subfertile beef heifers; Table S2. Differentially expressed genes (90) between the fertile and subfertile groups (*p*-value < 0.05 and |log2 fc| > 0.5). The genes up- or down regulated were reported in subfertile heifers; Table S3. The genes correlated with 90 differentially expressed genes identified with the PCIT approach, with a correlation > 0.95; Table S4. Network analysis of genes co-expressed with differentially expressed genes in fertile and subfertile groups. The hub genes for each group are highlighted in bold. The hub targets exclusive for each group are highlighted in bold and italics; Table S5. Central reference network including a fertile and subfertile network constructed using 7244 nodes. The genes highlighted in bold have a z-score of + 1.96; the connectivity is reported as gain or loss in the subfertile group; Table S6. Differentially abundant metabolites (9) between the fertile and subfertile groups (*p*-value < 0.05). Metabolites that were high/low in abundance were represented by the direction of log2FC in the subfertile group; Table S7. The metabolites correlated with 9 differentially abundant metabolites identified with the PCIT approach, with a correlation >0.80; Table S8. Network analysis of metabolites co-expressed with differentially abundant metabolites in fertile and subfertile groups. The hub metabolites for each group are highlighted in bold. The hub targets exclusive to each group are highlighted in bold and italics; Table S9. Central reference network including a fertile and subfertile metabolite network constructed using 111 nodes. The metabolites highlighted in bold have a z-score of +1.96; the connectivity is reported as gain or loss in the subfertile group; Table S10. Correlated gene–metabolite pairs identified with the PCIT approach, with a correlation > 0.95; Table S11. Central reference network including significant fertile and subfertile gene–metabolite pairs; Table S12. Pathways over-represented by the significant gene–metabolite pairs in the fertile and subfertile groups; Table S13. List of primers to amplify significant DEGs in the fertile and subfertile groups.
